# *r*-2,*c*-6-Bis(4-chloro­phen­yl)-*c*-3,*t*-3-dimethyl­piperidin-4-one

**DOI:** 10.1107/S1600536808036325

**Published:** 2008-11-13

**Authors:** S. S. Ilango, S. Ponnuswamy, P. Gayathri, A. Thiruvalluvar, R. J. Butcher

**Affiliations:** a Department of Chemistry, Government Arts College (Autonomous), Coimbatore 641 018, Tamilnadu, India; b PG Research Department of Physics, Rajah Serfoji Government College (Autonomous), Thanjavur 613 005, Tamilnadu, India; c Department of Chemistry, Howard University, 525 College Street NW, Washington, DC 20059, USA

## Abstract

In the title mol­ecule, C_19_H_19_Cl_2_NO, the piperidine ring adopts a chair conformation and the dihedral angle between the two benzene rings is 77.23 (7)°. In the crystal structure, mol­ecules are linked by N—H⋯O and C—H⋯O hydrogen bonds, and a weak C—H⋯π inter­action is also observed.

## Related literature

For a related crystal structure, see: Gayathri *et al.* (2008 ▶). For background on the biological activities of piperidones, see: Dimmock *et al.* (2001 ▶); Perumal *et al.* (2001 ▶). For the synthesis and stereodynamics of piperidin-4-ones and their derivatives, see: Ponnuswamy *et al.* (2002 ▶). For the synthesis, see: Noller & Baliah (1948 ▶).
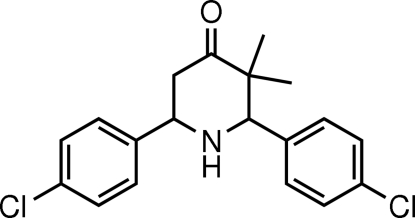


## Experimental

### 

#### Crystal data

C_19_H_19_Cl_2_NO
*M* *_r_* = 348.25
Orthorhombic, 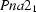

*a* = 13.1627 (5) Å
*b* = 22.4739 (7) Å
*c* = 5.8794 (2) Å
*V* = 1739.23 (10) Å^3^
*Z* = 4
Mo *K*α radiation
μ = 0.38 mm^−1^
*T* = 200 (2) K
0.44 × 0.31 × 0.22 mm

#### Data collection

Oxford Diffraction Gemini R diffractometer
Absorption correction: multi-scan (*CrysAlis RED*; Oxford Diffraction, 2008 ▶) *T*_min_ = 0.950, *T*_max_ = 1.000 (expected range = 0.874–0.920)
19147 measured reflections
5694 independent reflections
2460 reflections with *I* > 2σ(*I*)
*R*_int_ = 0.051

#### Refinement

*R*[*F* ^2^ > 2σ(*F* ^2^)] = 0.039
*wR*(*F* ^2^) = 0.083
*S* = 0.82
5694 reflections
212 parameters
1 restraint
H atoms treated by a mixture of independent and constrained refinement
Δρ_max_ = 0.34 e Å^−3^
Δρ_min_ = −0.31 e Å^−3^
Absolute structure: Flack (1983 ▶), 2278 Friedel pairs
Flack parameter: −0.03 (5)

### 

Data collection: *CrysAlis CCD* (Oxford Diffraction, 2008 ▶); cell refinement: *CrysAlis RED* (Oxford Diffraction, 2008 ▶); data reduction: *CrysAlis RED*; program(s) used to solve structure: *SHELXS97* (Sheldrick, 2008 ▶); program(s) used to refine structure: *SHELXL97* (Sheldrick, 2008 ▶); molecular graphics: *ORTEP-3* (Farrugia, 1997 ▶); software used to prepare material for publication: *PLATON* (Spek, 2003 ▶).

## Supplementary Material

Crystal structure: contains datablocks global, I. DOI: 10.1107/S1600536808036325/hb2832sup1.cif

Structure factors: contains datablocks I. DOI: 10.1107/S1600536808036325/hb2832Isup2.hkl

Additional supplementary materials:  crystallographic information; 3D view; checkCIF report

## Figures and Tables

**Table 1:** Hydrogen-bond geometry (Å, °)

*D*—H⋯*A*	*D*—H	H⋯*A*	*D*⋯*A*	*D*—H⋯*A*
N1—H1⋯O4^i^	0.853 (17)	2.312 (17)	3.092 (2)	152.3 (15)
C23—H23⋯O4^ii^	0.95	2.56	3.377 (2)	144
C31—H31*B*⋯*Cg*1^iii^	0.98	2.96	3.7265 (15)	136

Symmetry codes: (i) 
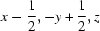
; (ii) 
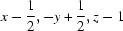
; (iii) 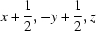. *Cg*1 is the centroid of the C61–C66 ring.

**Fig. 1.**
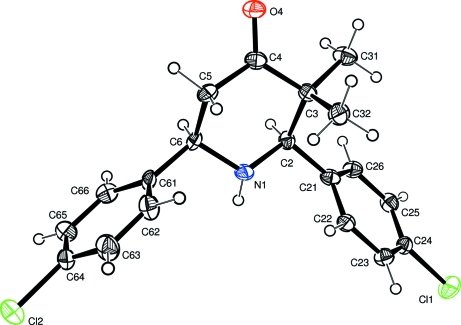
The molecular structure of (I), showing displacement ellipsoids drawn at the 30% probability level. H atoms are shown as small spheres of arbitrary radius.

**Fig. 2.**
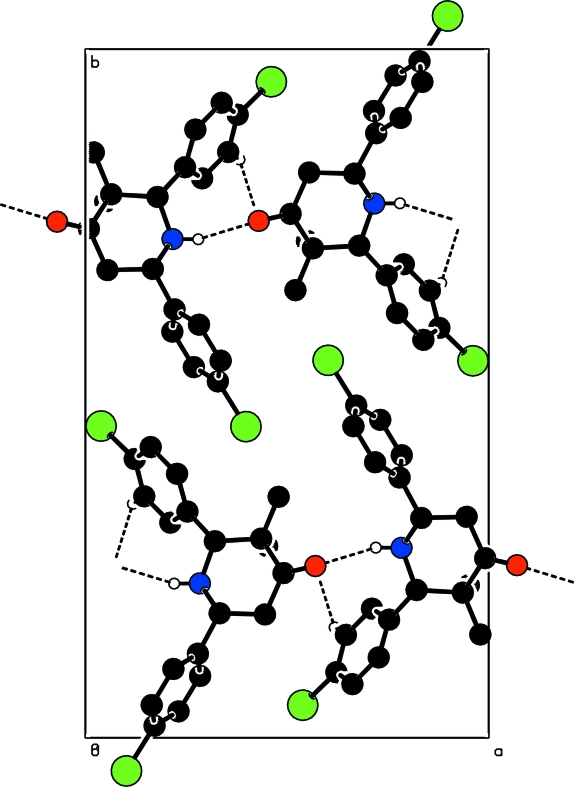
The packing of (I), viewed down the c axis. Dashed lines indicate hydrogen bonds. H atoms not involved in hydrogen bonding have been omitted.

## supplementary crystallographic information

### Comment

Piperidones are an important group of heterocyclic compounds in the field of medicinal chemistry due to their biological activities, including cytotoxic and anticancer properties (Dimmock *et al.*, 2001). Piperidones were also reported to possess analgesic, anti-inflammatory, central nervous system (*CNS*), local anaesthetic, anticancer and antimicrobial activity (Perumal *et al.*, 2001). The design and synthesis of conformationally anchored molecules is an important approach towards improving potency and selectivity. One such class of compounds constitutes piperidin-4-ones and their derivatives, whose synthesis and stereodynamics are well investigated (Ponnuswamy *et al.*, 2002). The crystal structure of r-2,c-6-Bis(4-chlorophenyl)-t-3-isopropyl-1-nitrosopiperidin-4-one has been reported, wherein the piperidine ring adopts a chair conformation (Gayathri *et al.*, 2008).

In the title molecule, C_19_H_19_Cl_2_NO (Fig. 1), the piperidine ring adopts a chair conformation. The phenyl rings at position 2,6 and one of the methyl groups attached to the piperidine ring in 3, have equatorial orientations. The dihedral angle between the two phenyl rings is 77.23 (7)°. In the crystal, the molecules are linked by N1—H1···O4 (*x* - 1/2, 1/2 - *y*, *z*) and C23—H23···O4(*x* - 1/2, -*y* + 1/2, *z* - 1) hydrogen bonds (Table 1). Further, a C31—H31B···π interaction involving the phenyl ring (C61—C66) at position 6 also present in the crystal structure.

### Experimental

The procedure adopted for the preparation of the title heterocyclic compound is similar to that of Noller & Baliah (1948). Ammonium acetate (7.7 g, 0.1 mol), 4-chlorobenzaldehyde (28.1 g, 0.2 mol) and 3-methyl-2-butanone (10.7 ml, 0.1 mol) were dissolved in 70 ml of rectified spirit. The resulting solution was heated to boiling and set aside for a day. The oily base obtained was converted into its hydrochloride by the addition of concentrated hydrochloric acid and the separated solid was filtered. Then the hydrochloride was neutralized with liquid ammonia. The resulting solid was filtered and purified by recrystallization from ethanol to yield colourless plates of (I). The yield of the product obtained was 28.65 g (82%).

### Refinement

Atom H1 attached to N1 was located in a difference fourier map and refined isotropically. The remaining H atoms were positioned geometrically and allowed to ride on their parent atoms, with C—H = 0.95, 0.98, 0.99 and 1.00 Å for C*sp*^2^, methyl, methylene and methine C, respectively; *U*_iso_(H) = k*U*_eq_(C), where k = 1.5 for methyl and 1.2 for all other H atoms.

### Crystal data

**Table:**

C_19_H_19_Cl_2_NO	*D*_x_ = 1.330 Mg m^−^^3^
*M**_r_* = 348.25	Melting point: 402(1) K
Orthorhombic, *P**n**a*2_1_	Mo *K*α radiation λ = 0.71073 Å
Hall symbol: P 2c -2n	Cell parameters from 4389 reflections
*a* = 13.1627 (5) Å	θ = 4.5–32.5º
*b* = 22.4739 (7) Å	µ = 0.38 mm^−^^1^
*c* = 5.8794 (2) Å	*T* = 200 (2) K
*V* = 1739.23 (10) Å^3^	Rectangular-plate, colourless
*Z* = 4	0.44 × 0.31 × 0.22 mm
*F*_000_ = 728	

### Data collection

**Table:**

Oxford Diffraction R Gemini diffractometer	5694 independent reflections
Radiation source: fine-focus sealed tube	2460 reflections with *I* > 2σ(*I*)
Monochromator: graphite	*R*_int_ = 0.052
Detector resolution: 10.5081 pixels mm^-1^	θ_max_ = 32.5º
*T* = 200(2) K	θ_min_ = 4.7º
φ and ω scans	*h* = −18→19
Absorption correction: multi-scan(CrysAlis RED; Oxford Diffraction, 2008)	*k* = −33→33
*T*_min_ = 0.950, *T*_max_ = 1.000	*l* = −8→8
19147 measured reflections	

### Refinement

**Table:**

Refinement on *F*^2^	Hydrogen site location: difmap and geom
Least-squares matrix: full	H atoms treated by a mixture of independent and constrained refinement
*R*[*F*^2^ > 2σ(*F*^2^)] = 0.039	*w* = 1/[σ^2^(*F*_o_^2^) + (0.0371*P*)^2^] where *P* = (*F*_o_^2^ + 2*F*_c_^2^)/3
*wR*(*F*^2^) = 0.083	(Δ/σ)_max_ = 0.001
*S* = 0.82	Δρ_max_ = 0.34 e Å^−^^3^
5694 reflections	Δρ_min_ = −0.31 e Å^−^^3^
212 parameters	Extinction correction: none
1 restraint	Absolute structure: Flack (1983), 2278 Friedel pairs
Primary atom site location: structure-invariant direct methods	Flack parameter: −0.03 (5)
Secondary atom site location: difference Fourier map	

### Special details

**Table:**

Geometry. Bond distances, angles *etc*. have been calculated using the rounded fractional coordinates. All su's are estimated from the variances of the (full) variance-covariance matrix. The cell e.s.d.'s are taken into account in the estimation of distances, angles and torsion angles
Refinement. Refinement of *F*^2^ against ALL reflections. The weighted *R*-factor *wR* and goodness of fit *S* are based on *F*^2^, conventional *R*-factors *R* are based on *F*, with *F* set to zero for negative *F*^2^. The threshold expression of *F*^2^ > 2σ(*F*^2^) is used only for calculating *R*-factors(gt) *etc*. and is not relevant to the choice of reflections for refinement. *R*-factors based on *F*^2^ are statistically about twice as large as those based on *F*, and *R*- factors based on ALL data will be even larger.

### Fractional atomic coordinates and isotropic or equivalent isotropic displacement parameters (Å^2^)

**Table:**

	*x*	*y*	*z*	*U*_iso_*/*U*_eq_	
Cl1	0.03898 (4)	0.45263 (2)	0.55799 (11)	0.0585 (2)	
Cl2	0.10182 (5)	−0.04812 (2)	1.04537 (12)	0.0655 (2)	
O4	0.57022 (11)	0.24936 (6)	1.2121 (2)	0.0424 (5)	
N1	0.28387 (13)	0.22418 (6)	1.0183 (3)	0.0312 (5)	
C2	0.32122 (13)	0.28540 (7)	1.0528 (3)	0.0277 (5)	
C3	0.43587 (14)	0.28927 (8)	0.9833 (3)	0.0307 (6)	
C4	0.49124 (15)	0.23934 (9)	1.1093 (3)	0.0341 (6)	
C5	0.44531 (14)	0.17905 (9)	1.1022 (4)	0.0411 (7)	
C6	0.33289 (15)	0.18081 (8)	1.1659 (3)	0.0321 (6)	
C21	0.25298 (15)	0.32842 (8)	0.9275 (3)	0.0312 (6)	
C22	0.21068 (13)	0.31249 (8)	0.7180 (3)	0.0314 (6)	
C23	0.14602 (14)	0.35069 (8)	0.6055 (3)	0.0345 (6)	
C24	0.12245 (15)	0.40493 (8)	0.7019 (3)	0.0352 (6)	
C25	0.16202 (16)	0.42206 (8)	0.9082 (3)	0.0377 (7)	
C26	0.22727 (14)	0.38327 (8)	1.0207 (3)	0.0347 (6)	
C31	0.47926 (14)	0.34992 (8)	1.0480 (4)	0.0439 (7)	
C32	0.45062 (15)	0.27850 (10)	0.7274 (3)	0.0426 (7)	
C61	0.27905 (14)	0.12205 (8)	1.1398 (3)	0.0305 (6)	
C62	0.28440 (17)	0.08976 (9)	0.9400 (3)	0.0439 (7)	
C63	0.23098 (19)	0.03820 (9)	0.9104 (4)	0.0504 (8)	
C64	0.17155 (15)	0.01751 (8)	1.0837 (4)	0.0424 (7)	
C65	0.16451 (18)	0.04755 (10)	1.2868 (4)	0.0461 (8)	
C66	0.21822 (16)	0.09989 (10)	1.3129 (3)	0.0432 (8)	
H1	0.2194 (13)	0.2237 (7)	1.032 (3)	0.023 (5)*	
H2	0.31631	0.29460	1.21887	0.0333*	
H5A	0.48191	0.15262	1.20910	0.0493*	
H5B	0.45276	0.16236	0.94724	0.0493*	
H6	0.32639	0.19436	1.32722	0.0385*	
H22	0.22667	0.27498	0.65262	0.0376*	
H23	0.11790	0.33976	0.46270	0.0414*	
H25	0.14517	0.45957	0.97251	0.0452*	
H26	0.25477	0.39444	1.16384	0.0416*	
H31A	0.44241	0.38120	0.96623	0.0659*	
H31B	0.55139	0.35148	1.00700	0.0659*	
H31C	0.47187	0.35603	1.21217	0.0659*	
H32A	0.41564	0.30973	0.64133	0.0639*	
H32B	0.42235	0.23962	0.68637	0.0639*	
H32C	0.52328	0.27930	0.69113	0.0639*	
H62	0.32636	0.10380	0.81998	0.0527*	
H63	0.23513	0.01699	0.77096	0.0605*	
H65	0.12350	0.03259	1.40682	0.0553*	
H66	0.21343	0.12114	1.45215	0.0519*	

### Atomic displacement parameters (Å^2^)

**Table:**

	*U*^11^	*U*^22^	*U*^33^	*U*^12^	*U*^13^	*U*^23^
Cl1	0.0547 (3)	0.0505 (3)	0.0703 (4)	0.0179 (3)	−0.0132 (4)	0.0120 (3)
Cl2	0.0681 (4)	0.0421 (3)	0.0864 (4)	−0.0195 (3)	−0.0183 (4)	0.0074 (3)
O4	0.0273 (8)	0.0530 (9)	0.0468 (8)	−0.0024 (7)	−0.0046 (8)	0.0054 (7)
N1	0.0184 (9)	0.0324 (8)	0.0427 (10)	0.0015 (7)	−0.0010 (8)	0.0036 (7)
C2	0.0273 (10)	0.0267 (9)	0.0292 (9)	0.0009 (8)	0.0047 (10)	0.0017 (10)
C3	0.0262 (11)	0.0338 (10)	0.0321 (10)	−0.0012 (9)	0.0015 (9)	0.0010 (8)
C4	0.0225 (10)	0.0414 (12)	0.0385 (11)	0.0026 (9)	0.0030 (10)	−0.0003 (9)
C5	0.0293 (11)	0.0359 (11)	0.0581 (14)	0.0052 (9)	−0.0109 (11)	0.0094 (11)
C6	0.0329 (11)	0.0306 (10)	0.0328 (9)	0.0049 (9)	−0.0053 (9)	0.0087 (8)
C21	0.0223 (10)	0.0374 (12)	0.0339 (10)	−0.0015 (9)	0.0035 (9)	−0.0045 (9)
C22	0.0337 (11)	0.0273 (10)	0.0331 (10)	−0.0018 (9)	−0.0029 (10)	−0.0017 (8)
C23	0.0307 (11)	0.0362 (10)	0.0366 (11)	0.0023 (9)	−0.0043 (9)	0.0008 (9)
C24	0.0306 (11)	0.0327 (11)	0.0424 (11)	0.0012 (9)	−0.0018 (10)	0.0117 (10)
C25	0.0354 (12)	0.0304 (11)	0.0472 (11)	0.0037 (10)	0.0028 (11)	−0.0011 (10)
C26	0.0282 (10)	0.0364 (10)	0.0394 (10)	0.0017 (9)	−0.0005 (10)	−0.0045 (9)
C31	0.0355 (11)	0.0386 (11)	0.0577 (12)	−0.0024 (9)	−0.0058 (13)	−0.0094 (11)
C32	0.0360 (13)	0.0550 (14)	0.0368 (11)	−0.0003 (10)	0.0049 (11)	0.0074 (11)
C61	0.0304 (11)	0.0246 (9)	0.0364 (10)	0.0045 (9)	−0.0026 (9)	0.0077 (8)
C62	0.0537 (15)	0.0387 (12)	0.0393 (11)	−0.0057 (11)	0.0080 (11)	0.0021 (10)
C63	0.0657 (17)	0.0362 (13)	0.0493 (12)	−0.0036 (12)	−0.0009 (14)	−0.0095 (11)
C64	0.0390 (12)	0.0308 (10)	0.0573 (14)	−0.0048 (9)	−0.0099 (13)	0.0126 (12)
C65	0.0384 (14)	0.0485 (14)	0.0514 (13)	−0.0074 (11)	−0.0026 (11)	0.0076 (11)
C66	0.0445 (14)	0.0455 (14)	0.0397 (11)	−0.0003 (12)	−0.0064 (11)	0.0015 (10)

### Geometric parameters (Å, °)

**Table:**

Cl1—C24	1.7528 (19)	C62—C63	1.367 (3)
Cl2—C64	1.7518 (19)	C63—C64	1.366 (3)
O4—C4	1.223 (2)	C64—C65	1.375 (3)
N1—C2	1.475 (2)	C65—C66	1.381 (3)
N1—C6	1.456 (2)	C2—H2	1.0000
N1—H1	0.853 (17)	C5—H5A	0.9900
C2—C3	1.566 (3)	C5—H5B	0.9900
C2—C21	1.511 (2)	C6—H6	1.0000
C3—C31	1.526 (3)	C22—H22	0.9500
C3—C32	1.536 (3)	C23—H23	0.9500
C3—C4	1.529 (3)	C25—H25	0.9500
C4—C5	1.484 (3)	C26—H26	0.9500
C5—C6	1.527 (3)	C31—H31A	0.9800
C6—C61	1.507 (3)	C31—H31B	0.9800
C21—C22	1.398 (3)	C31—H31C	0.9800
C21—C26	1.391 (3)	C32—H32A	0.9800
C22—C23	1.378 (3)	C32—H32B	0.9800
C23—C24	1.380 (3)	C32—H32C	0.9800
C24—C25	1.375 (3)	C62—H62	0.9500
C25—C26	1.391 (3)	C63—H63	0.9500
C61—C66	1.387 (3)	C65—H65	0.9500
C61—C62	1.383 (3)	C66—H66	0.9500
			
C2—N1—C6	113.25 (15)	N1—C2—H2	108.00
C6—N1—H1	112.1 (11)	C3—C2—H2	108.00
C2—N1—H1	109.3 (11)	C21—C2—H2	108.00
N1—C2—C21	109.36 (14)	C4—C5—H5A	109.00
N1—C2—C3	109.70 (14)	C4—C5—H5B	109.00
C3—C2—C21	114.21 (14)	C6—C5—H5A	109.00
C2—C3—C31	110.20 (14)	C6—C5—H5B	109.00
C2—C3—C4	106.98 (14)	H5A—C5—H5B	108.00
C4—C3—C32	107.37 (15)	N1—C6—H6	109.00
C31—C3—C32	109.73 (16)	C5—C6—H6	109.00
C4—C3—C31	110.87 (15)	C61—C6—H6	109.00
C2—C3—C32	111.63 (15)	C21—C22—H22	120.00
O4—C4—C5	121.87 (18)	C23—C22—H22	120.00
O4—C4—C3	120.61 (17)	C22—C23—H23	120.00
C3—C4—C5	117.52 (16)	C24—C23—H23	120.00
C4—C5—C6	111.36 (16)	C24—C25—H25	121.00
N1—C6—C5	107.50 (15)	C26—C25—H25	121.00
C5—C6—C61	114.09 (16)	C21—C26—H26	119.00
N1—C6—C61	108.52 (15)	C25—C26—H26	119.00
C2—C21—C26	121.30 (16)	C3—C31—H31A	109.00
C2—C21—C22	120.15 (16)	C3—C31—H31B	109.00
C22—C21—C26	118.50 (17)	C3—C31—H31C	109.00
C21—C22—C23	120.61 (17)	H31A—C31—H31B	109.00
C22—C23—C24	119.48 (17)	H31A—C31—H31C	109.00
C23—C24—C25	121.64 (17)	H31B—C31—H31C	109.00
Cl1—C24—C25	119.47 (14)	C3—C32—H32A	109.00
Cl1—C24—C23	118.88 (14)	C3—C32—H32B	109.00
C24—C25—C26	118.54 (17)	C3—C32—H32C	109.00
C21—C26—C25	121.22 (17)	H32A—C32—H32B	109.00
C62—C61—C66	117.66 (18)	H32A—C32—H32C	109.00
C6—C61—C62	121.51 (17)	H32B—C32—H32C	109.00
C6—C61—C66	120.76 (16)	C61—C62—H62	119.00
C61—C62—C63	121.81 (19)	C63—C62—H62	119.00
C62—C63—C64	119.2 (2)	C62—C63—H63	120.00
Cl2—C64—C65	119.33 (17)	C64—C63—H63	120.00
Cl2—C64—C63	119.38 (17)	C64—C65—H65	121.00
C63—C64—C65	121.28 (19)	C66—C65—H65	121.00
C64—C65—C66	118.7 (2)	C61—C66—H66	119.00
C61—C66—C65	121.31 (18)	C65—C66—H66	119.00
			
C6—N1—C2—C3	64.86 (19)	N1—C6—C61—C62	68.3 (2)
C6—N1—C2—C21	−169.16 (15)	N1—C6—C61—C66	−108.6 (2)
C2—N1—C6—C5	−64.02 (19)	C5—C6—C61—C62	−51.5 (2)
C2—N1—C6—C61	172.13 (15)	C5—C6—C61—C66	131.6 (2)
N1—C2—C3—C4	−51.75 (18)	C2—C21—C22—C23	178.11 (17)
N1—C2—C3—C31	−172.37 (15)	C26—C21—C22—C23	0.8 (3)
N1—C2—C3—C32	65.44 (19)	C2—C21—C26—C25	−178.05 (17)
C21—C2—C3—C4	−174.92 (14)	C22—C21—C26—C25	−0.8 (3)
C21—C2—C3—C31	64.5 (2)	C21—C22—C23—C24	−0.5 (3)
C21—C2—C3—C32	−57.7 (2)	C22—C23—C24—Cl1	−179.30 (14)
N1—C2—C21—C22	−36.1 (2)	C22—C23—C24—C25	0.1 (3)
N1—C2—C21—C26	141.12 (18)	Cl1—C24—C25—C26	179.33 (15)
C3—C2—C21—C22	87.3 (2)	C23—C24—C25—C26	−0.1 (3)
C3—C2—C21—C26	−95.5 (2)	C24—C25—C26—C21	0.4 (3)
C2—C3—C4—O4	−132.07 (17)	C6—C61—C62—C63	−176.1 (2)
C2—C3—C4—C5	47.7 (2)	C66—C61—C62—C63	0.9 (3)
C31—C3—C4—O4	−11.9 (2)	C6—C61—C66—C65	176.77 (19)
C31—C3—C4—C5	167.88 (17)	C62—C61—C66—C65	−0.2 (3)
C32—C3—C4—O4	107.97 (19)	C61—C62—C63—C64	−0.8 (3)
C32—C3—C4—C5	−72.3 (2)	C62—C63—C64—Cl2	179.29 (17)
O4—C4—C5—C6	129.70 (19)	C62—C63—C64—C65	0.0 (3)
C3—C4—C5—C6	−50.1 (2)	Cl2—C64—C65—C66	−178.65 (17)
C4—C5—C6—N1	53.9 (2)	C63—C64—C65—C66	0.6 (3)
C4—C5—C6—C61	174.28 (16)	C64—C65—C66—C61	−0.5 (3)

### Hydrogen-bond geometry (Å, °)

**Table:**

*D*—H···*A*	*D*—H	H···*A*	*D*···*A*	*D*—H···*A*
N1—H1···O4^i^	0.853 (17)	2.312 (17)	3.092 (2)	152.3 (15)
C23—H23···O4^ii^	0.95	2.56	3.377 (2)	144
C31—H31B···Cg1^iii^	0.98	2.96	3.7265 (15)	136

Symmetry codes: (i) *x*−1/2, −*y*+1/2, *z*; (ii) *x*−1/2, −*y*+1/2, *z*−1; (iii) *x*+1/2, −*y*+1/2, *z*.
